# Optic Pathway Glioma in Adults: A Systematic Review and Individual Patient-Level Analysis of Clinical Characteristics and Prognostic Factors

**DOI:** 10.3390/cancers18081225

**Published:** 2026-04-13

**Authors:** Daniel O’Connor, Hanyu Qiu, Kishore Balasubramanian, Ruoqi Ye, Christopher S. Graffeo, Michael J. Feldman, Karl E. Balsara

**Affiliations:** 1 Department of Neurosurgery, University of Oklahoma College of Medicine, Oklahoma City, OK 73104, USA; daniel-a-oconnor@ou.edu (D.O.); hanyu-qiu@ou.edu (H.Q.); kishore.b@tamu.edu (K.B.); ruoqi-ye@ou.edu (R.Y.); christopher-graffeo@ou.edu (C.S.G.); michael-feldman@ou.edu (M.J.F.); 2 College of Medicine, Texas A&M Health Science Center, Houston, TX 77030, USA

**Keywords:** adult glioma, adult optic pathway glioma, optic pathway glioma, systematic review

## Abstract

**Objective**: Optic pathway glioma (OPG) diagnosed in adults represents a rare and understudied tumor subtype. While pediatric OPGs are typically benign and associated with *NF1* and *RAS*/*MAPK* pathway dysregulation, less is known about the clinical characteristics and prognostic drivers of OPGs in adults. **Methods**: A systematic review was conducted in accordance with PRISMA guidelines across multiple databases. Studies reporting patient-level data and follow-up for patients diagnosed with optic pathway glioma at age ≥ 18 years were included. **Results**: Ninety-six studies comprising 149 adult patients were analyzed. Median patient age was 47 years (range: 18–90), and 51.0% of tumors were high-grade (WHO grade 3–4). Increasing age at diagnosis was significantly correlated with higher WHO grade (ρ = 0.600, *p* < 0.001), and optic tract involvement was associated with high-grade disease (χ^2^ = 8.08, *p* = 0.004; ϕ = 0.26). Median follow-up was 12 months, with 74 patients alive and 75 deceased at last follow-up. WHO grade was strongly associated with overall survival (log-rank *p* < 0.0001), with 24-month survival ranging from 96.9% for grade 1 tumors to 11.3% for grade 4 tumors. Compared with observation or steroid-only management, both surgical and non-surgical oncologic treatments were associated with longer observed survival, although no significant difference was observed between active treatment modalities. **Conclusions**: Optic pathway gliomas in adults exhibit a multimodal biologic distribution, encompassing both indolent low-grade tumors and aggressive high-grade malignancies. Survival outcomes appear to be primarily driven by tumor biology, with age and anatomic involvement correlating with tumor grade. Prospective, multicenter studies with comprehensive molecular profiling are needed to refine prognostic stratification and guide evidence-based management of this rare disease.

## Simple Summary

Optic pathway gliomas diagnosed in adults are rare central nervous system tumors that differ in important ways from the common pediatric form of the disease. Because most published evidence consists of isolated case reports and small case series, there is limited consensus regarding their clinical characteristics and prognostic stratification. To address this gap, we performed a systematic review with individual patient-level analysis of published cases. Our findings suggest that many adult-diagnosed cases exhibit high-grade pathology, while a subset of younger adults had slower-growing tumors resembling pediatric-type disease. Survival analyses demonstrated strong associations between tumor grade and outcomes. Active treatment approaches, including surgical and non-surgical therapies, were associated with longer observed survival compared with supportive care alone; however, causal treatment effects cannot be inferred from the available literature. These findings help clarify the landscape of this rare disease while highlighting the need for prospective, multicenter investigation with more consistent molecular characterization.

## 1. Introduction

Optic pathway gliomas (OPGs) are glial tumors that arise within the visual pathway and may occur along the optic nerve (ON), optic chiasm (OC), or optic tract (OT) [1,2]. OPGs predominantly affect younger patients, with the vast majority presenting in childhood [3,4]. Epidemiologic studies have demonstrated that more than 80% of OPGs arise in pediatric patients, most commonly in children under the age of 10, and collectively account for approximately 3–5% of all pediatric central nervous system (CNS) tumors [3,5]. In the pediatric setting, OPGs are typically indolent, slow-growing, low-grade tumors often associated with neurofibromatosis type 1 (NF1) and *RAS/MAPK* pathway dysregulation [5,6]. *NF1*-independent cases frequently harbor *BRAF* fusions, resulting in overactivation of *RAF/MEK* signaling [5,6]. Syndromic pediatric-onset OPGs are commonly managed with careful radiographic observation, with some lesions demonstrating spontaneous regression over time [5]. Treatment is generally reserved for cases exhibiting progressive tumor growth or clinically significant visual impairment [5].

In contrast to pediatric cases, OPGs diagnosed in adults often show markedly different clinical behavior [7]. In adult cases, OPGs are more frequently reported as malignant, with an aggressive clinical course and a higher prevalence of high-grade tumors [7]. Visual deterioration in this setting is often rapid, progressing over weeks, with tumor-related mortality occurring within months of diagnosis [7]. Unlike pediatric OPGs, the molecular landscape of adult OPGs is not well defined [5]. No definitive treatment protocols exist for high-grade adult OPGs [5,7]. Multimodal strategies incorporating radiation, chemotherapy, supportive care, and surgical intervention have been employed; however, outcomes remain heterogeneous and the overall prognosis is poor [5,7].

Due to their rarity, optic pathway gliomas in adults have not been well characterized through prospective investigation [5]. The existing literature is fragmented, consisting largely of isolated case reports and small case series, which limits the ability to define epidemiologic patterns, biologic behavior, and prognostic factors unique to this population. To address this gap, we performed a systematic review with individual patient-level analysis to comprehensively synthesize reported OPG cases that were diagnosed in adulthood. Our objectives were to characterize demographic and tumor features, evaluate anatomic correlates of disease behavior, and identify prognostic factors associated with survival outcomes. By consolidating the available evidence, this study aims to establish a clearer baseline for understanding OPGs in adults and inform future efforts in risk stratification and clinical decision-making, and provide a foundation for future prospective investigation of this rare and understudied entity.

## 2. Methods

### 2.1. Study Selection

A systematic literature search was conducted in accordance with the Preferred Reporting Items for Systematic Reviews and Meta-Analyses (PRISMA) guidelines [8] (Supplementary Table S2). This study was conducted as a systematic review with individual patient-level analysis of published studies. A formal protocol was not prospectively registered prior to study initiation.

PubMed, Embase, and Ovid MEDLINE databases were searched from database inception through 3 December 2025, using the following Boolean search strategy: [(“optic nerve” OR “optic pathway”) AND “glioma” AND (“adult” OR “adults”)]. All records were imported into Rayyan (https://help.rayyan.ai/hc/en-us/articles/4406419348369-What-is-the-version-of-Rayyan, accessed on 9 April 2026) for screening, and duplicate entries were manually removed.

Studies were included if they: (1) reported patients aged ≥18 years with a confirmed diagnosis of optic pathway glioma; (2) provided individual patient-level clinical, treatment, outcome, and follow-up data; and (3) were published in English. Adult status was defined based on age at diagnosis (≥18 years), as the timing of true tumor onset could not be reliably determined from the available literature. Studies were excluded if they: (1) were autopsy reports, animal studies, or did not address clinical management; (2) were literature reviews, commentaries, perspectives, or editorials; (3) lacked patient-level data; or (4) were non-English or non-peer-reviewed sources. 

Two independent reviewers (D.O. and R.Y.) screened all titles and abstracts and subsequently assessed full-text articles meeting inclusion criteria. To prevent inclusion of duplicate cases, studies were assessed for potential overlap based on author groups, institutional affiliations, patient demographics, tumor characteristics, and reporting timeframes. When overlap was suspected, the most comprehensive report was retained. Disagreements were resolved by a third reviewer (H.Q.).

### 2.2. Data Extraction and Statistical Analysis

Two reviewers (D.O. and R.Y.) extracted data from each article, which was confirmed independently by one additional reviewer (H.Q.). Extracted data included paper authors, year published, patient demographics, clinical presentation, treatment details, and survival outcomes. For case series in which only a subset of patients met the inclusion criteria (patients ≥18 years with confirmed optic pathway glioma), data were extracted only for the eligible cases. For each study, two independent authors (H.Q. and D.O.) assessed the risk of bias by applying the Joanna Briggs Institute checklists for case reports and case series. The level of evidence of included studies was evaluated using the Joanna Briggs Institute Levels of Evidence guidelines. 

Microsoft Excel (Microsoft Corporation, Redmond, WA, USA) was used for data collection and descriptive analyses. Continuous variables are reported as medians with ranges, and categorical variables as frequencies with percentages. Descriptive data were handled using available-case analysis, with each variable analyzed using the subset of patients for whom data were reported. As a result, denominators varied across variables depending on data availability. 

Survival analyses were performed using Jamovi (The Jamovi Project, open source, version 2.6.26.0), including the “ClinicoPathDescriptives” and “jsurvival” packages. Overall survival (OS) was defined as the time from initial treatment to death or last follow-up. Progression-free survival (PFS) was defined as the time from initial treatment to reported progression or recurrence, as defined by the source study. Patients without an event were censored at the time of last follow-up. Due to variability in reporting across studies, definitions of progression were not standardized.

Cox proportional hazards modeling was performed to evaluate factors associated with overall survival. Covariates included age at diagnosis, WHO grade, optic tract involvement, treatment category, and patient sex. WHO grade was dichotomized into low-grade (WHO grade 1–2) and high-grade (WHO grade 3–4) categories to maximize the number of patients included in the model, as some source studies reported tumor grade only as “low-grade” or “high-grade” without specifying the exact WHO grade. Because the dataset was derived from published case reports and case series rather than a single independent cohort, observations may not have been fully independent, and potential clustering of patients within studies was not explicitly modeled. No formal sensitivity or robustness analyses were performed given the limited sample size and heterogeneity of the dataset, which precluded meaningful alternative model analyses. Proportional hazards assumptions were not formally tested given these constraints.

To address treatment-selection heterogeneity, treatment exposure was modeled as a three-level categorical variable (observation/steroid only, surgical treatment, and non-surgical treatment), with observation-only management serving as the reference category. Patients who only received a biopsy were not considered to have received a surgical oncologic treatment, and were classified according to subsequent management, either in the non-surgical treatment group if they received oncologic therapy, or in the observation/steroid-only group if no active oncologic treatment was administered. A secondary Cox model restricted to patients receiving active treatment was performed to compare surgical versus non-surgical treatment directly, using identical covariates. Additional analysis included Kaplan–Meier survival estimates, chi-squared testing, and Spearman’s rank correlation. Statistical significance was defined as *p* < 0.05.

## 3. Results

### 3.1. Study Selection

**Figure 1:**
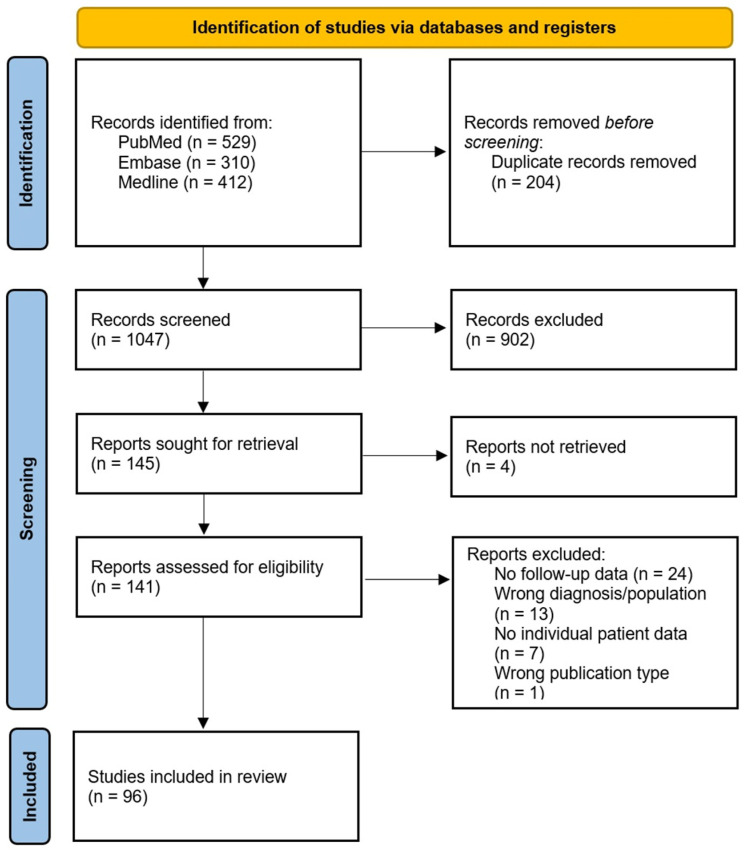
PRISMA flow diagram of the literature screening.

Figure 1 summarizes the study selection process using a PRISMA flow diagram. The initial systematic search identified a total of 1251 studies (PubMed: 529, Embase: 310, MEDLINE: 412). After removal of 204 duplicates, 1047 studies were screened using predefined inclusion and exclusion criteria. Finally, 96 studies which consisted of 30 case series and 66 case reports, representing Joanna Briggs Institute (JBI) levels of evidence 4.c and 4.d, respectively, were included in the final synthesis [9,10,11,12,13,14,15,16,17,18,19,20,21,22,23,24,25,26,27,28,29,30,31,32,33,34,35,36,37,38,39,40,41,42,43,44,45,46,47,48,49,50,51,52,53,54,55,56,57,58,59,60,61,62,63,64,65,66,67,68,69,70,71,72,73,74,75,76,77,78,79,80,81,82,83,84,85,86,87,88,89,90,91,92,93,94,95,96,97,98,99,100,101,102,103,104]. Included studies, level of evidence, and risk-of-bias assessments are summarized in Table 1.

Among case series, 24 of 30 studies (80.0%) were classified as low risk of bias, while 6 (20.0%) were moderate risk. The most frequently unmet or unclear criteria involved consecutive inclusion and completeness of inclusion, reflecting limitations in reporting of patient selection methods. In contrast, case reports demonstrated consistently low risk of bias, with 63 of 66 studies (95.5%) classified as low risk and 3 (4.5%) as moderate risk. The most commonly missing domain in case reports was the reporting of adverse events or complications.

### 3.2. Patient and Case Characteristics

The included cohort comprised a total of 149 patients with optic pathway glioma diagnosed in adulthood. Individual patient characteristics including age, sex, tumor grade, and follow-up are summarized in Supplemental Table S1. The median age of the cohort was 47 years (range: 18–90 years), with 79 male patients (53.0%) and 70 female patients (47.0%). Patient characteristics including presenting symptoms, duration of symptoms prior to presentation, lesion location, adjacent structure invasion, and tumor histologic classifications are summarized in Table 2. The cohort demonstrated a near-equal sex distribution (53.0% male, 47.0% female). Vision loss was the most common presenting symptom, reported in 99.3% of cases, with a relatively even distribution between unilateral and bilateral involvement. Tumors most frequently involved the optic nerve (73.2%) and optic chiasm (60.4%), with less frequent optic tract involvement (16.8%).

### 3.3. Clinical Management

Median follow-up duration of the cohort was 12 months (range: 0.1–420 months). 74 patients (49.7%) were alive at last reported follow-up, while 75 patients (50.3%) were deceased. Tumor and treatment characteristics including WHO grade, primary treatment, adjuvant therapy, postoperative complications, tumor progression, symptom assessment at last follow-up, and duration of progression-free survival are summarized in Table 3. High-grade tumors (WHO grade 3–4) comprised a substantial proportion of the cohort; however, a notable subset of patients had low-grade disease (WHO grade 1–2). Surgical intervention was the most frequently utilized primary treatment modality (54.4%, 81/149 patients), while non-surgical oncologic therapies were also commonly employed (34.9%, 52/149 patients). Radiation-based approaches accounted for the majority of non-surgical treatment, delivered either as radiation alone (18.1%) or combined chemoradiotherapy (12.1%).

**Figure 2:**
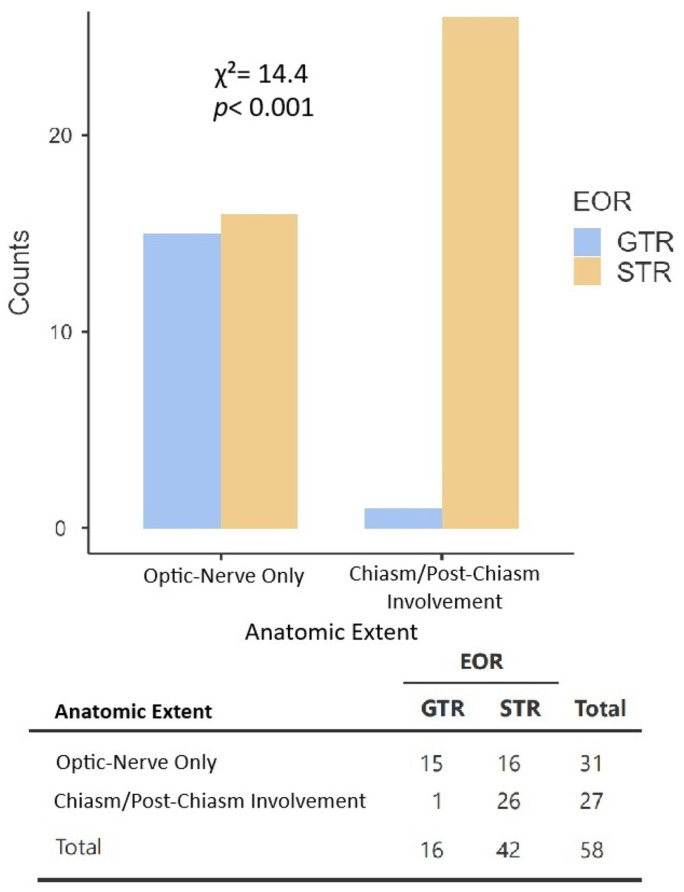
Association between extent of resection (EOR) and tumor anatomic involvement. Gross total resection (GTR) was significantly more likely to be achieved in tumors confined to the optic nerve compared to tumors with chiasm/post-chiasm involvement (*χ*^2^ = 14.4, *p* < 0.001; *ϕ* = 0.50). Abbreviations: GTR = gross total resection, STR = subtotal resection, EOR = extent of resection.

The association between extent of resection (EOR) and tumor anatomic involvement was assessed, with findings displayed in Figure 2. Among surgical cases with reported EOR (n = 58), gross total resection (GTR) was more frequently achieved in tumors confined to the optic nerve (48.4%) compared with tumors exhibiting chiasmal or post-chiasmal involvement (3.7%). This difference in EOR was statistically significant (*χ*^2^ = 14.4, *p* < 0.001; *ϕ* = 0.50). 

### 3.4. Molecular Findings

26 patient cases reported molecular findings. Case characteristics including patient age, tumor location, and WHO grade are summarized along with identified molecular findings in Table 4. *NF1* and *TP53* alterations were the most frequently reported molecular features in this cohort, with additional alterations including *PTEN* mutations, *CDKN2A/B* deletions, and *TERT* mutations observed in smaller subsets.

### 3.5. Survival Analysis

**Figure 3:**
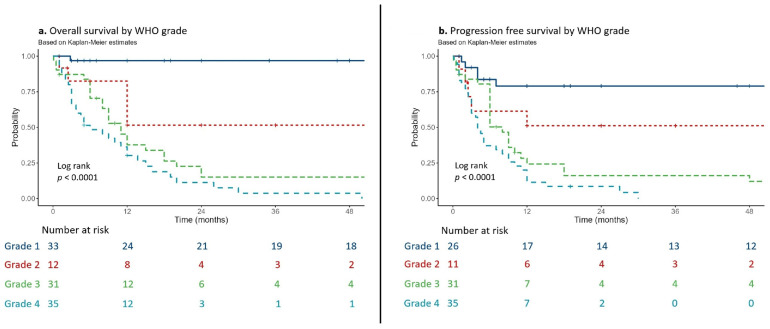
Survival data for the cohort. (**a**). Kaplan–Meier curve for overall survival by WHO grade. (**b**). Kaplan–Meier curve for progression-free survival by WHO grade.

Kaplan–Meier estimates of overall survival (OS) and progression-free survival (PFS) stratified by WHO grade are shown in Figure 3. OS estimate at 12 months was 96.9% (95% CI: 91.0–100%) for grade 1 cases, 51.6% (95% CI: 28.3–93.9%) for grade 2 cases, 37.7% (95% CI: 23.4–60.7%) for grade 3 cases, and 30.3% (95% CI: 18.1–50.5%) for grade 4 cases. At 24 months, OS estimates were 96.9% (95% CI: 91.0–100%) for grade 1, 51.6% (95% CI: 28.3–93.9%) for grade 2, 15.1% (95% CI: 6.2–36.9%) for grade 3, and 11.3% (95% CI: 4.0–31.8%) for grade 4. The difference in OS between WHO grades was statistically significant (log-rank *p* < 0.0001). 

Kaplan–Meier estimates of PFS at 12 months were 79.0% (95% CI: 64.1–97.3%) for grade 1, 51.1% (95% CI: 27.9–93.6%) for grade 2, 24.2% (95% CI: 12.5–47.0%) for grade 3, and 11.4% (95% CI: 4.5–28.7%) for grade 4. PFS estimates at 24 months were 79.0% (95% CI: 64.1–97.3%) for grade 1, 51.1% (95% CI: 27.9–93.6%) for grade 2, 16.2% (95% CI: 6.8–38.6%) for grade 3, and 8.6% (95% CI: 2.9–25.3%) for grade 4. The PFS difference between WHO grades was statistically significant (log-rank *p* < 0.0001). 

A multivariable Cox proportional hazards model of overall survival was constructed including patient age, WHO grade, optic tract involvement, treatment category, and patient sex. In this primary model, increasing age at diagnosis (*p* < 0.001) and higher WHO grade (*p* = 0.005) were independently associated with worse overall survival. Relative to observation/steroid-only management, both surgical treatment (HR 0.30, 95% CI 0.13–0.70) and non-surgical oncologic treatment (HR 0.36, 95% CI 0.17–0.78) were associated with longer observed survival. Cox model parameters for the primary model, including hazard ratios, 95% confidence intervals, *p* values, and model metrics, are summarized in Table 5.

A secondary Cox model restricted to patients receiving active treatment was performed to directly compare surgical versus non-surgical treatment modalities. This model incorporated the same covariates as the primary model. In this analysis, no significant difference in overall survival was observed between surgical and non-surgical treatment approaches (HR 0.85, 95% CI 0.47–1.52, *p* = 0.583).

### 3.6. Clinical Correlates of WHO Grade

**Figure 4:**
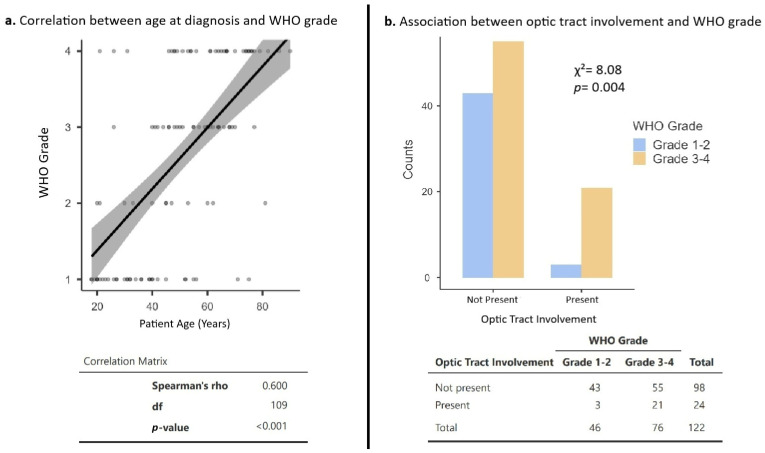
Correlates with WHO grade in adult optic pathway gliomas. (**a**). Scatterplot showing a positive correlation between age at diagnosis and WHO grade (Spearman’s *ρ* = 0.600, df = 109, *p* < 0.001). (**b**). Distribution of low grade (WHO grade 1–2) versus high grade (WHO grade 3–4) tumors by optic tract involvement, demonstrating a significant association between optic tract involvement and higher WHO grade (*χ*^2^ = 8.08, *p* = 0.004; *ϕ* = 0.26).

Spearman rank correlation analysis demonstrated a statistically significant positive association between higher age at diagnosis and increased tumor WHO grade (Spearman’s ρ = 0.600, df = 109, *p* < 0.001) (Figure 4a). Optic tract involvement was also associated with WHO grade, with tumors involving the optic tract more frequently classified as WHO grade 3–4 compared with tumors without optic tract involvement (χ^2^ = 8.08, *p* = 0.004; ϕ = 0.26). Among cases with optic tract involvement, 21 out of 24 tumors (87.5%) were WHO grade 3–4, whereas 55 out of 98 tumors (56.1%) without optic tract involvement were WHO grade 3–4 (Figure 4b).

## 4. Discussion

Optic pathway glioma in adults is a rare and uncommonly reported disease, and the available literature is correspondingly limited and heterogeneous. In the present analysis, the evidence base consisted primarily of case reports and small case series, with risk-of-bias assessment demonstrating generally strong reporting quality among individual reports but greater variability among case series. These characteristics are important to consider when interpreting the findings of this study. Accordingly, the results should be viewed as providing a descriptive overview of observed patterns in this population, rather than definitive conclusions, particularly with respect to treatment-related associations.

### 4.1. Patient and Tumor Characteristics

A defining feature of this adult-only cohort was the high prevalence of malignant disease, with 51.3% of patients diagnosed with WHO grade 3–4 tumors (21.3% grade 3, 23.3% grade 4, 6.7% unspecified high-grade), in stark contrast to pediatric-focused and pediatric-dominant mixed series where high-grade optic pathway glioma (OPG) is exceedingly rare [105,106]. A recent mixed-cohort OPG meta-analysis reported grade 3, grade 4, and unspecified high-grade tumors each accounting for less than 1% of cases, underscoring the profound divergence in tumor grade distribution between OPGs diagnosed in pediatrics compared with those diagnosed in adults [106].

Within this adult population, age emerged as the primary correlate of tumor grade. As demonstrated in Figure 4a, increasing age at diagnosis was significantly associated with higher WHO grade (Spearman’s ρ = 0.600, *p* < 0.001), a pattern consistent with broader adult glioma literature where advancing age independently correlates with higher-grade, more aggressive disease, including in the post-2021 WHO classification era [107,108]. Notably, the relationship observed here was not uniform across ages. Low-grade tumors clustered predominantly in younger adults, whereas higher-grade tumors were increasingly concentrated at older ages. This relatively segmented, multimodal distribution suggests that at least a subset of low-grade adult OPGs may share biological similarity with pediatric-type disease, potentially representing late-presenting or slowly progressive lesions that retain indolent pediatric-type characteristics into adulthood.

Beyond age, the anatomic origin and area of tumor involvement may further refine risk stratification. Some prior reports and series describing OPGs in adults have suggested that tumors involving or arising from the posterior optic pathway, especially beyond the chiasm, may be associated with more aggressive clinical behavior and worse outcomes [1,3,63,109]. Consistent with these observations, we found that optic tract involvement was significantly associated with high-grade disease (WHO Grade 3–4) in our cohort, with 87.5% of tumors involving the optic tract classified as high-grade, compared with 56.1% of tumors without optic tract involvement (χ^2^ = 8.08, *p* = 0.004; Figure 4b). Notably, although a significant association between anatomic involvement and WHO grade was observed, optic tract involvement did not retain independent prognostic significance for overall survival when incorporated into multivariable Cox proportional hazards modeling alongside tumor grade, suggesting that posterior optic pathway involvement does not appear to confer additional prognostic information beyond its association with higher-grade disease within the present cohort.

Although an association between tumor location and grade was observed in the available cohort, limitations in sample size and data heterogeneity preclude definitive conclusions regarding a direct biologic mechanism linking posterior optic pathway involvement to high-grade disease and aggressive tumor behavior. Further studies will be essential to identify the biologic basis of this observation. Nevertheless, anatomic features of tumor involvement may still provide meaningful contextual information in clinical practice when considered alongside other pathologic and clinical findings. Taken together, our data highlights the biologic variability in adult optic pathway gliomas, encompassing both indolent, low-grade tumors in younger adults and aggressive, high-grade malignancies in older patients. This demographic and anatomic overview provides a framework for contextualizing disease risk at presentation and refining prognostic expectations.

### 4.2. Clinical Management

Management strategies for pediatric optic pathway gliomas are typically conservative, often centering on observation in the absence of visual deterioration, with escalation of therapy guided by patient age, symptom progression, and tumor behavior [2,5]. In contrast, adult cases in this cohort were more frequently managed with surgical intervention and/or radiotherapy, consistent with the substantially higher prevalence of high-grade disease. Overall, clinical management strategies for optic pathway gliomas in adult patients were highly heterogeneous, reflecting both biologic variability and the absence of a definitive treatment approach.

Surgery was the most common primary treatment modality in our cohort, utilized in 54.4% of cases (81/149 patients). Surgical intervention most often resulted in subtotal resection, with gross total resection achieved infrequently. This pattern is consistent with surgical experiences in pediatric OPG, as the anatomic location of OPGs is generally not amenable to gross total resection [2]. Aggressive surgical resection is associated with substantial risks including visual, endocrine, and vascular morbidity, and is therefore pursued selectively [2]. However, when extent of resection was examined in relation to tumor anatomy, tumors confined to the optic nerve achieved gross total resection more frequently than tumors demonstrating chiasmal or post-chiasmal extension. The effect size of this association in our cohort was moderate to large (*ϕ* = 0.50), consistent with potentially greater technical feasibility for complete resection in anatomically isolated anterior optic pathway disease.

Radiation therapy, either alone or in combination with chemotherapy, was employed in a substantial subset of patients (30.2%, 45/149 cases). Chemotherapy alone and observation were less frequently reported, underscoring both the high proportion of malignant disease and a tendency toward aggressive upfront management in adults compared with pediatric practice [5,7]. The variability in treatment selection across studies highlights the individualized nature of decision-making in this population and reflects ongoing uncertainty regarding optimal management strategies for OPGs in adult patients [5,7].

### 4.3. Survival and Outcomes

Survival outcomes in this cohort demonstrated a wide range of clinical trajectories, reflecting the segmented, multimodal distribution of optic pathway glioma biology in adult patients. Kaplan–Meier analyses revealed substantially different overall survival and progression-free survival profiles across WHO grades, with low-grade tumors exhibiting low mortality and prolonged survival, while high-grade tumors were associated with rapid clinical decline. These findings reinforce the biologic variability within our adult OPG cohort and demonstrate the fundamentally different natural histories of indolent low-grade versus aggressive high-grade OPGs. Notably, the WHO grade 1 tumors in our cohort demonstrated overall survival and progression-free survival patterns closely resembling pediatric-onset counterparts [110,111]. When considered alongside age-at-onset trends, these observations support the possibility that a subset of low-grade adult OPGs may share biologic features with pediatric-type disease.

Beyond tumor grade, age at diagnosis emerged as an independent prognostic factor in multivariable Cox proportional hazards analysis. Each additional year of age was associated with an approximate 4% increase in the hazard of death (HR 1.04 per year, *p* < 0.001), even after adjustment for WHO grade, anatomic involvement, treatment modality, and sex. This finding is consistent with findings in broader adult glioma literature, in which advancing age is associated with worse outcomes and is thought to reflect additional biologic or host-related influences not fully captured by WHO grade alone [112]. 

With respect to primary treatment approach on outcomes, both surgical and non-surgical treatment strategies were associated with statistically significant longer observed survival compared with observation or steroid-only management. However, multivariable analysis did not identify a clearly superior treatment modality among patients receiving active intervention, and no significant difference in overall survival was observed between surgical and non-surgical approaches when directly compared. These findings do not imply therapeutic equivalence. Rather, these observations underscore the absence of a standardized approach for optic pathway glioma management in adult patients and reflect the substantial heterogeneity in current treatment strategies. Management decisions appear to remain highly individualized and be shaped by institutional practice and clinical circumstance rather than definitive field consensus. These findings should be interpreted as observational associations rather than evidence of causal treatment benefit. Given the retrospective and heterogeneous nature of the included literature, confounding by indication and publication bias are both important considerations, as patients selected for surgical or oncologic treatment may differ systematically from those managed conservatively. Accordingly, treatment effects cannot be definitively inferred from the available data.

Taken together, these observations suggest that current survival outcomes of adult patients with optic pathway glioma appear to be primarily driven by tumor biology, with patient age and anatomic involvement serving as important correlates of disease severity, rather than by any clearly identifiable primary treatment approach. The absence of a clearly superior primary management strategy highlights both the limitations of the existing literature and the ongoing uncertainty that characterizes adult OPG care. These findings underscore the need for improved biologic characterization, more consistent reporting, and prospective data collection to strengthen prognostic stratification and support continued development of evidence-based management strategies for this uncommon and clinically challenging tumor population.

### 4.4. Molecular Observations

Molecular profiling was reported for a limited subset of adult optic pathway gliomas in this study (n = 26), reflecting the predominance of older case reports and series in the literature as well as inconsistent testing practices. Accordingly, molecular findings in this cohort should only be interpreted as descriptive and hypothesis-generating, rather than as definitive indicators. Nevertheless, several recurring patterns align with established glioma biology and may help contextualize the biologic heterogeneity observed in adult OPG behavior. 

Consistent with pediatric OPG literature, *NF1* alterations in our cohort were often observed in younger patients and were associated with WHO grade 1 tumors [113]. High-grade *NF1*-associated tumors were uncommon; however, when present, they were accompanied by additional oncogenic alterations, most notably involving *TP53*. This pattern aligns with prior clinical and experimental evidence suggesting that *NF1* loss alone is typically insufficient for malignant transformation, and that progression to high-grade disease often requires cooperating genetic events [114,115].

Beyond *NF1*-associated cases, the molecular alterations observed in high-grade adult optic pathway gliomas were more consistent with broader adult glioma biology [116,117]. Alterations involving *TP53* and other components of the p53 pathway, including *MDM2* amplification and *CDKN2A* loss, were observed among high-grade tumors in our cohort, alongside additional features classically associated with aggressive gliomas such as *PTEN* mutation [116,117,118]. Although available molecular data is limited, these preliminary observations suggest that high-grade OPGs in adult patients may share developmental and molecular pathways more aligned with other adult gliomas than with pediatric-type OPGs, despite arising from a similar anatomic pathway. 

Given the inconsistency and limited quantity of available data, conclusions regarding molecular characterization cannot be drawn. Importantly, the absence of systematic molecular profiling across the included studies limits the ability to fully contextualize these findings within contemporary glioma molecular frameworks. Increasing evidence suggests that molecular features, including gene expression profiles and pathway-level alterations, may outperform traditional clinical variables in predicting tumor behavior and patient outcomes in broader glioma populations [119,120,121]. Accordingly, future investigations of optic pathway gliomas in adults should prioritize integration of molecular profiling, including transcriptomic and epigenetic characterization, into clinical datasets. Prospective molecular-focused investigation will be essential for refining prognostic stratification and advancing therapeutic strategies in this rare and complex patient population.

### 4.5. Limitations

This systematic review and individual patient analysis of optic pathway gliomas in adult patients has several limitations that should be considered when interpreting the findings. Due to the rarity of adult OPGs, the included literature consisted primarily of case reports and small case series, which are inherently susceptible to selection, reporting, and publication biases. Long-term outcomes were incompletely reported in many studies, limiting assessment of late progression, treatment-related morbidity, and long-term outcomes, particularly for low-grade tumors with indolent courses. In addition, key clinical variables were inconsistently described across reports, which may have introduced unmeasured confounding into survival analyses. Treatment approach, particularly in cases managed with observation or supportive care, likely reflects underlying disease severity and clinical status, introducing confounding by indication that limits causal interpretation of survival differences across management strategies. 

This historical cohort also spans eras preceding the 2021 WHO classification update, during which tumor grading relied predominantly on histopathologic criteria. As a result, the incorporation of molecular features into contemporary diagnostic frameworks raises the possibility that tumor grade assignment across the included studies may not be fully concordant with current WHO criteria, and that some tumors may be classified differently under modern standards. Furthermore, molecular profiling was available for only a limited subset of patients, reflecting historical variability in testing practices and precluded prognostic analysis of molecular findings. Prospective cohorts with standardized reporting, comprehensive molecular characterization, and long-term follow-up will be required to validate the observations in this study.

## 5. Conclusions

Optic pathway gliomas (OPGs) diagnosed in adults are a rare and biologically heterogeneous tumor population that differs substantially from pediatric disease. In this individual patient-level analysis, adult OPGs demonstrated a high prevalence of aggressive, high-grade tumors associated with poor outcomes, while a distinct subset followed an indolent clinical course resembling pediatric-type OPGs. Increasing age at diagnosis and posterior optic pathway involvement emerged as key correlates of higher-grade disease, with current survival outcomes driven largely by tumor biology and patient age.

A wide range of treatment approaches were reported, with both surgical and non-surgical oncologic treatments associated with longer observed survival over observation or supportive care. Nonetheless, direct comparison of surgical and non-surgical approaches did not demonstrate a clear survival advantage of one strategy over the other, reflecting the substantial heterogeneity and ongoing variability that characterize adult optic pathway glioma management in clinical practice and the existing literature. Limited molecular observations suggest that OPGs in adults encompass a spectrum of molecular profiles, ranging from pediatric-type low-grade patterns to features more aligned with high-grade adult gliomas, despite a shared anatomic origin. Collectively, our findings highlight the need for prospective, multicenter collaboration with comprehensive molecular profiling and standardized reporting to refine prognostic stratification and optimize management strategies for this uncommon and biologically variable patient population.

## Figures and Tables

**Table 1:** JBI risk of bias assessment.

JBI Risk of Bias for Included Case Series (JBI Level of Evidence 4.c)																		
Study Author	Year	Q1	Q2		Q3		Q4		Q5	Q6	Q7	Q8		Q9		Q10	% Yes	Risk
Li et al. [3]	2023	√	√		√		√		U	√	√	√		√		U	80	Low
Shoji et al. [23]	2020	√	√		√		√		U	√	√	√		√		√	90	Low
Bin Abdulqader et al. [26]	2018	√	√		√		U		U	√	√	√		√		√	80	Low
Alireza et al. [29]	2017	√	√		√		U		U	√	√	√		√		X	70	Moderate
Borghei-Razavi et al. [32]	2016	√	√		√		√		U	√	√	√		√		X	80	Low
Traber et al. [37]	2015	√	√		√		U		U	√	√	√		√		√	80	Low
Bilgin et al. [40]	2014	√	√		√		X		X	√	√	√		√		√	80	Low
Caignard et al. [41]	2014	√	√		√		U		X	√	√	√		√		√	80	Low
Theeler et al. [47]	2014	√	√		U		√		√	√	√	√		√		√	90	Low
Shriver et al. [52]	2012	√	√		U		√		√	√	√	√		√		√	90	Low
Wu-Chen et al. [58]	2009	√	√		√		U		X	√	√	√		U		X	70	Moderate
Sharif et al. [62]	2006	√	√		√		U		√	√	√	√		√		√	90	Low
Danesh-Meyer et al. [63]	2005	√	√		√		U		X	√	√	√		√		√	80	Low
Kwon et al. [64]	2005	√	√		U		U		X	√	√	√		√		√	70	Moderate
Wulc et al. [81]	1989	√	√		√		X		U	√	√	√		√		√	80	Low
Albers et al. [82]	1988	√	√		√		X		X	√	√	√		√		√	80	Low
Svitra et al. [85]	1986	X	√		√		X		X	√	√	√		√		X	60	Moderate
Horwich et al. [86]	1985	√	√		√		U		U	√	√	√		√		√	80	Low
Borit et al. [88]	1982	√	√		√		U		U	√	√	√		√		√	80	Low
Kupersmith et al. [90]	1981	√	√		√		U		U	√	√	√		√		√	80	Low
Dosoretz et al. [91]	1980	√	√		√		U		U	√	√	√		√		√	80	Low
Spoor et al. [92]	1980	√	√		√		X		X	√	√	√		√		√	80	Low
Wright et al. [93]	1980	√	√		√		U		U	√	√	√		√		X	70	Moderate
Enoksson et al. [94]	1978	X	√		√		U		X	√	√	√		√		√	70	Moderate
Harper et al. [95]	1978	√	√		√		U		X	√	√	√		√		√	80	Low
Harter et al. [96]	1978	√	√		√		U		U	√	√	√		√		√	80	Low
Lowes et al. [97]	1978	√	√		√		U		U	√	√	√		√		√	80	Low
Miller et al. [99]	1974	√	√		√		√		√	√	√	√		√		X	90	Low
Hoyt et al. [102]	1973	√	√		√		X		X	√	√	√		√		√	80	Low
Spencer [103]	1972	X	√		√		X		X	√	√	√		√		X	60	Moderate
Total Studies: 30	Total %:	90%	100%		90%		20%		13%	100%	100%	100%		97%		73%		
JBI Risk of Bias for Included Case Reports (JBI Level of Evidence 4.d)																		
Study Author	Year	Q1		Q2		Q3		Q4		Q5	Q6		Q7		Q8		% Yes	Risk
Peyton et al. [9]	2025	√		√		√		√		√	√		X		√		87.5	Low
Kassotis et al. [10]	2024	√		√		√		√		√	√		√		√		100	Low
Magharious et al. [11]	2024	√		√		√		√		√	√		U		√		87.5	Low
Ng et al. [12]	2024	√		√		√		√		√	√		X		√		87.5	Low
Amisaki et al. [13]	2023	√		√		√		√		√	√		√		√		100	Low
Dung et al. [14]	2023	√		√		√		√		√	√		X		√		87.5	Low
Mulhem [16]	2023	√		√		√		√		√	√		√		√		100	Low
Cao et al. [17]	2022	√		√		√		√		√	√		X		√		87.5	Low
Sun et al. [18]	2022	√		√		√		√		√	√		U		√		87.5	Low
Prado et al. [19]	2021	√		√		√		√		√	√		√		√		100	Low
Heiland et al. [20]	2020	√		√		√		√		√	√		X		√		87.5	Low
Hong et al. [21]	2020	√		√		√		√		√	√		√		√		100	Low
Ramakrishnan et al. [22]	2020	√		√		√		√		√	√		X		√		87.5	Low
Bayley et al. [24]	2019	√		√		√		√		√	√		X		√		87.5	Low
Lv et al. [25]	2019	√		√		√		√		√	√		√		√		100	Low
Mastorakos et al. [27]	2018	√		√		√		√		√	√		X		√		87.5	Low
Wang et al. [28]	2018	√		√		√		√		√	√		X		√		87.5	Low
Lin et al. [30]	2017	√		√		√		√		√	√		X		√		87.5	Low
Menon et al. [31]	2017	√		√		√		√		√	√		X		√		87.5	Low
Cimino et al. [33]	2016	√		√		√		√		√	√		X		√		87.5	Low
Lyapichev et al. [34]	2016	√		√		√		√		√	√		X		√		87.5	Low
Nagaishi et al. [35]	2015	√		√		√		√		√	√		X		√		87.5	Low
Nagia et al. [36]	2015	√		√		√		√		√	√		X		√		87.5	Low
Arrese et al. [38]	2014	√		√		√		√		√	√		X		√		87.5	Low
Bhaker et al. [39]	2014	√		√		√		√		√	√		X		√		87.5	Low
Colpak et al. [42]	2014	√		√		√		√		√	√		X		√		87.5	Low
Della Puppa et al. [43]	2014	√		√		√		√		√	√		X		√		87.5	Low
Kim [44]	2014	√		√		√		√		√	√		X		√		87.5	Low
Pecen et al. [45]	2014	√		√		√		√		√	√		X		√		87.5	Low
Sarkar et al. [46]	2014	√		√		√		√		√	√		X		√		87.5	Low
Ashur-Fabian et al. [48]	2013	√		√		√		√		√	√		√		√		100	Low
Jiang et al. [49]	2013	√		√		√		√		X	√		X		√		75	Moderate
Liu et al. [50]	2013	√		√		√		√		√	√		X		√		87.5	Low
Manojlovic Gacic et al. [51]	2012	√		√		√		√		√	√		X		√		87.5	Low
Matloob et al. [53]	2011	√		√		√		√		√	√		X		√		87.5	Low
Simao et al. [54]	2011	√		√		√		√		√	√		X		√		87.5	Low
Chacko et al. [55]	2010	√		√		√		√		√	√		X		√		87.5	Low
Pasol et al. [56]	2010	√		√		√		√		√	√		X		√		87.5	Low
Kawasaki [57]	2009	√		√		√		√		√	√		X		√		87.5	Low
Abou-Zeid et al. [59]	2008	√		√		√		√		√	√		X		√		87.5	Low
Dinh et al. [60]	2007	√		√		√		√		√	√		X		√		87.5	Low
Miyamoto et al. [61]	2006	√		√		√		√		√	√		√		√		100	Low
Albayrak et al. [65]	2004	√		√		√		√		√	√		X		√		87.5	Low
Chernov et al. [66]	2004	√		√		√		√		√	√		X		√		87.5	Low
Wabbels et al. [67]	2004	√		√		√		√		√	√		X		√		87.5	Low
Murphy et al. [68]	2003	√		√		√		√		√	√		X		√		87.5	Low
Yokoyama et al. [69]	2003	√		√		√		√		√	√		X		√		87.5	Low
Synowitz et al. [70]	2002	√		√		√		√		√	√		X		√		87.5	Low
Colosimo et al. [71]	1999	√		√		√		√		√	√		X		√		87.5	Low
Dario et al. [72]	1999	√		√		√		√		√	√		X		√		87.5	Low
Friedman et al. [73]	1998	√		U		√		√		X	X		X		√		50	Moderate
Brodovsky et al. [74]	1997	√		√		√		√		√	√		√		√		100	Low
Liauw et al. [75]	1996	√		√		√		√		√	√		X		√		87.5	Low
Lim et al. [76]	1996	√		√		√		√		√	√		√		√		100	Low
Millar et al. [77]	1995	√		√		√		√		√	√		X		√		87.5	Low
Woiciechowsky et al. [78]	1995	√		√		√		√		√	√		X		√		87.5	Low
Taphoorn et al. [79]	1989	√		√		√		√		√	√		X		√		87.5	Low
Topliss [80]	1989	√		√		√		√		√	√		X		√		87.5	Low
Hufnagel et al. [83]	1988	√		√		√		√		√	√		X		√		87.5	Low
Ramani et al. [84]	1988	√		√		√		√		√	√		X		√		87.5	Low
Barbaro et al. [87]	1982	√		√		√		√		√	√		X		√		87.5	Low
Shapiro et al. [89]	1982	√		√		√		√		√	√		√		√		100	Low
Manor et al. [98]	1976	√		√		√		√		X	√		X		√		75	Moderate
Gibberd et al. [100]	1973	√		√		√		√		X	√		√		√		87.5	Low
Hamilton et al. [101]	1973	√		√		√		√		√	√		√		√		100	Low
Otenasek et al. [104]	1968	√		√		√		√		√	√		X		√		87.5	Low
Total Studies: 66	Total %:	100%		98%		100%		100%		94%	98%		20%		100%			

Questions for JBI Level of Evidence 4.c: Q1—Were there clear criteria for inclusion in the case series? Q2—Was the condition measured in a standard, reliable way for all participants included in the case series? Q3—Were valid methods used for identification of the condition for all participants included in the case series? Q4—Did the case series have consecutive inclusion of participants? Q5—Did the case series have complete inclusion of participants? Q6—Was there clear reporting of the demographics of the participants in the study? Q7—Was there clear reporting of clinical information of the participants? Q8—Were the outcomes or follow-up results of cases clearly reported? Q9—Was there clear reporting of the presenting site(s)/clinic(s) demographic information? Q10—Was statistical analysis appropriate? Questions for JBI Level of Evidence 4.d: Q1—Were patient’s demographic characteristics clearly described? Q2—Was the patient’s history clearly described and presented as a timeline? Q3—Was the current clinical condition of the patient on presentation clearly described? Q4—Were diagnostic tests or methods and the results clearly described? Q5—Was the intervention(s) or treatment procedure(s) clearly described? Q6—Was the post-intervention clinical condition clearly described? Q7—Were adverse events (harms) or unanticipated events identified and described? Q8—Does the case report provide takeaway lessons? Abbreviations: √ = yes, U = unclear, X = no.

**Table 2:** Summary of patient characteristics.

Characteristic (No. of Patients for Whom Information Is Available)	Median or N	Range or %
Cohort size	149	
Demographics (n = 149)		
Age (years)	47	18–90
Gender (n = 149)		
Male	79	53.0
Female	70	47.0
Presenting symptoms/physical exam findings (n = 135) *		
Vision loss	134	99.3
Bilateral	67	49.6
Unilateral	65	48.1
Unspecified	2	1.5
Raised intracranial pressure	55	40.7
Visual field deficit	32	23.7
Eye/facial pain	18	13.3
Proptosis	15	11.1
Motor deficits	9	6.7
Color vision deficit	9	6.7
Swelling	3	2.2
Stabismus	1	0.7
Sensory deficit	1	0.7
Hallucinations	1	0.7
Dysphagia	1	0.7
Babinski response	1	0.7
Duration of symptoms prior to presentation (months) (n = 97)	2	0.07–156
Location of lesion (n = 149) *		
Optic nerve	109	73.2
Optic chiasm	90	60.4
Optic tract	25	16.8
Invasion of adjacent structures (n = 26) *		
Diencephalon	18	69.2
Cerebrum	9	34.6
Ventricular system	6	23.1
Midbrain region	3	11.5
Basal ganglia	2	7.7
Corpus callosum	1	3.8
Pituitary fossa	1	3.8
Histologic classification (n = 149)		
Glioblastoma	34	22.8
Low-grade astrocytic tumor	34	22.8
Anaplastic astrocytoma	33	22.1
Ganglioglioma	4	2.7
Gliosarcoma	2	1.3
Ependymoma	1	0.7
Unspecified glioma	41	27.5

* Some patients may fit multiple categories. Data represents the frequency of the individual findings in relation to the total sample size.

**Table 3:** Summary of tumor characteristics and clinical management.

Characteristic (No. of Patients for Whom Information Is Available)	Median or N	Range or %
Tumor WHO Grade (n = 149)		
Unspecified	38	25.5
High grade	10	6.7
Low grade	1	0.7
Grade 4	35	23.5
Grade 3	31	20.8
Grade 2	12	8.1
Grade 1	33	22.1
Primary treatment modality (n = 149)		
Surgical treatment	81	54.4
STR	42	28.2
GTR	16	10.7
Unspecified	23	15.4
Non-surgical treatment	52	34.9
Radiation	27	18.1
Chemoradiotherapy	18	12.1
Chemotherapy	7	4.7
Observation/steroid only	16	10.7
Adjuvant therapy after surgical resection (n = 73)		
None	31	42.5
Radiation	29	39.7
Chemoradiotherapy	11	15.1
Chemotherapy	2	2.7
Post-operative complications (n = 18) *		
Visual deterioration	7	38.9
Mental status decline	5	27.8
Focal motor deficits	5	27.8
Seizures	4	22.2
Endocrine disturbance	3	16.7
Ataxia	2	11.1
Incontinence	1	5.6
Tumor progression/recurrence (n = 96)		
Progression	49	51.0
None	44	45.8
Recurrence	3	3.1
Symptom assessment at last follow-up (n = 78)		
Worse	50	64.1
Improved	17	21.8
No change	11	14.1
Length of progression-free survival (months) (n = 130)	7.5	0.1–271.2
Length of last follow-up (months) (n = 149)	12	0.1–420
Survival status at last follow-up (n = 149)		
Dead	75	50.3
Alive	74	49.7

* Some patients had multiple post-operative complications. Data represents the frequency of the individual findings in relation to the total sample size. Abbreviations: STR = subtotal resection, GTR = gross total resection.

**Table 4:** Summary of patients with reported molecular alterations.

Study Author	Age (Years)	Tumor Location	WHO Grade *	Reported Molecular Alterations					
				*BRAF*	*NF1*	*MGMT*	*IDH1*	*TP53*	Other Reported Alterations
Wulc et al. [81]	31	ON, OC	1		Y				
Sharif et al. [62]	34	ON, OC	1		Y				
	21	ON, OC	1		Y				
	22	ON, OC	1		Y				
Manojlovic et al. [51]	55	ON	1		Y				
Ashur-Fabian et al. [48]	64	ON, OC, OT	4			Unmethylated			*PTEN* deletion, *1q36/19q13* loss
Bilgin et al. [40]	45	ON	2					Y	
Caignard et al. [41]	74	ON, OC	4				WT	Y	
	74	OC, OT	4				WT	Y	
Colpak et al. [42]	47	OC, OT	4				WT		
Theeler et al. [47]	27	OC	NOS		Y				
Nagaishi et al. [35]	64	ON, OC	3		Y	Unmethylated	WT	Y	
Nagia et al. [36]	81	ON	2				MT	Y	
Lyapichev et al. [34]	82	OC	4					Y	
Alireza et al. [29]	49	OC	4				WT		*EGFR* mutant
	67	ON, OC	4				WT		
	86	ON	4			Methylated	WT		
Mastorakos et al. [27]	66	ON	4			Methylated	WT	Y	
Heiland et al. [20]	71	ON	1						
Hong et al. [21]	39	ON, OC	1		Y				*FGFR1*, *PTPN11*
Ramakrishnan et al. [22]	48	ON, OC	4			Unmethylated	WT		
Sun et al. [18]	67	ON	4			Unmethylated	WT	Y	Amplified *MYC* and *CDK4*
Amisaki et al. [13]	66	ON	3		Y	Unmethylated	WT	Y	
Kassotis et al. [10]	35	ON	NOS	Y		Unmethylated			*CDKN2A/B* deletion
Magharious et al. [11]	46	OC	4				WT		*PTEN* mutation, *TERT* mutation, amplified *CDK4* and *MDM2*
Ng et al. [12]	73	ON	4				WT		*TERT* mutation, *CDKN2A* deletion
Total (n = 26): No. (%)				1 (3.8)	9 (34.6)	Unmethylated: 6 (23.1)	WT: 13 (50.0)	9 (34.6)	
						Methylated: 2 (7.7)	MT: 1 (3.8)		

* As reported in original study. Abbreviations: ON = optic nerve; OC = optic chiasm; OT = optic tract; NOS = not otherwise specified; Y = alteration present; WT = wild-type; MT = mutation. Background shading is used to group patients originating from the same study for clarity.

**Table 5:** Multivariable Cox model of overall survival.

Variable	Baseline	Comparison	HR	95% CI	*p*-Value
Age	N/A	Years	1.04	1.02–1.06	<0.001
WHO grade	Low Grade	High grade	5.72	2.19–14.93	0.005
Primary Treatment	Observation/steroid only	Surgical treatment	0.30	0.13–0.70	0.005
		Non-surgical treatment *	0.36	0.17–0.78	0.009
Optic tract involvement	Not present	Present	1.53	0.88–2.68	0.132
Sex	Male	Female	0.71	0.42–1.19	0.195

Model metrics: Number in model = 122; Number of events = 70; Likelihood ratio test = 92.635 (df = 6, *p* = 0.000). * Patients treated with radiation and/or chemotherapy without surgery were included in the non-surgical treatment group. Abbreviations: HR = hazard ratio, CI = confidence interval.

## Data Availability

All data generated or analyzed during this study are included in this article. Further inquiries may be directed to the corresponding author.

## Supplementary Materials

The following supporting information can be downloaded at: https://www.mdpi.com/article/10.3390/cancers18081225/s1, Table S1: Summary of included patients and studies. Abbreviations: Yr = year, N/A = not available, ON = optic nerve, OC = optic chiasm, OT = optic tract, PFS = progression-free survival, Mo = months, A = alive, D = deceased. Background shading is used to group patients originating from the same study for clarity. Table S2: PRISMA 2020 checklist.
