# Supplementary material for: Optic Pathway Glioma in Adults: A Systematic Review and Individual Patient-Level Analysis of Clinical Characteristics and Prognostic Factors
Source: Cancers (Basel). 2026 Apr 13;18(8):1225. doi: 10.3390/cancers18081225 (PMC13115507; doi:10.3390/cancers18081225)
Supplement: Supplementary file 1 [file cancers-18-01225-s001.zip › cancers-4194378 - Table S1.pdf]

Supplemental Table S1. Summary of included patients and studies

Study Author	Year	JBI Level of Evidence	Age (yr)	Sex	WHO Grade	Location	Primary Treatment	Progression	PFS (mo)	Last Follow-Up (mo)	Status
Peyton et al. [9]	2025	4.d	64	M	High Grade	OC	Chemoradiotherapy	Yes	3	3	D
Kassotis et al. [10]	2024	4.d	35	F	Low Grade	ON	Surgery	No	4	4	A
Magharious et al. [11]	2024	4.d	46	M	4	OC	Chemoradiotherapy	Yes	4	12	A
Ng et al. [12]	2024	4.d	73	M	4	ON	Observation/steroids only	Yes	2	2	D
Amisaki et al. [13]	2023	4.d	66	M	3	ON	Surgery	Yes	18	18	D
Dung et al. [14]	2023	4.d	46	M	3	OC, OT	Surgery	No	1	1	A
Li et al. [15]	2023	4.c	45	F	2	ON	Surgery	No	72	72	A
			36	F	1	ON	Surgery	No	87	87	A
Mulhem [16]	2023	4.d	77	F	4	OC	Surgery	No	19	19	D
Cao et al. [17]	2022	4.d	52	F	1	ON	Surgery	No	48	48	A
Sun et al. [18]	2022	4.d	67	M	4	ON	Observation/steroids only	Yes	5	5	A
Prado et al. [19]	2021	4.d	53	M	4	ON, OC	Chemotherapy	Yes	3.7	3.7	D
Heiland et al. [20]	2020	4.d	71	F	1	ON	Observation/steroids only	Yes	1.4	1.4	D
Hong et al. [21]	2020	4.d	39	F	1	ON, OC	Chemoradiotherapy	Yes	4	11	A
Ramakrishnan et al. [22]	2020	4.d	48	M	4	ON, OC	Chemoradiotherapy	Yes	7	12	D
Shoji et al. [23]	2020	4.c	18	M	1	OC	Surgery	Yes	N/A	132	A
			19	F	1	OC	Surgery	Yes	N/A	240	A
			26	F	1	OC	Surgery	Yes	2	60	A
			36	F	1	OC	Surgery	No	12	12	A
Bayley et al. [24]	2019	4.d	27	F	1	OC	Surgery	No	18	18	A
Lv et al. [25]	2019	4.d	61	F	4	ON	Chemoradiotherapy	Yes	15	26	D
Bin Abdulqader et al. [26]	2018	4.c	32	F	1	OC	Surgery	N/A	N/A	7	A
Mastorakos et al. [27]	2018	4.d	66	M	4	ON	Surgery	Yes	12	16	D
Wang et al. [28]	2018	4.d	30	M	2	OC	Surgery	No	12	12	A
Alireza et al. [29]	2017	4.c	49	M	4	OC	Chemotherapy	Yes	1	3	D
			67	F	4	ON, OC	Chemoradiotherapy	Yes	30	30	D
			86	F	4	ON	Chemotherapy	Yes	5	5	D
Lin et al. [30]	2017	4.d	21	M	4	ON, OC	Chemoradiotherapy	Yes	11	12	A
Menon et al. [31]	2017	4.d	40	M	1	ON	Surgery	No	1	1	A
Borghei-Razavi et al. [32]	2016	4.c	20	M	1	ON	Surgery	No	46	46	A
Cimino et al. [33]	2016	4.d	90	F	4	ON	Surgery	Yes	2.5	2.5	D
Lyapichev et al. [34]	2016	4.d	82	M	4	OC	Surgery	Yes	0.2	0.2	D
Nagaishi et al. [35]	2015	4.d	64	F	3	ON, OC	Radiation	No	10	10	A
Nagia et al. [36]	2015	4.d	81	F	2	ON	Observation/steroids only	N/A	N/A	6	A
Traber et al. [37]	2015	4.c	65	M	4	ON, OC	Chemoradiotherapy	Yes	4.5	4.5	D
			54	M	4	ON, OC, OT	Chemoradiotherapy	Yes	4	12	D
			64	F	3	ON, OC, OT	Radiation	Yes	6	6	D
			75	M	4	ON	Chemoradiotherapy	Yes	9	9	D
			76	F	4	ON, OC, OT	Radiation	Yes	3	3	D
Arrese et al. [38]	2014	4.d	30	F	1	OC, OT	Surgery	No	6	6	A
Bhaker et al. [39]	2014	4.d	27	M	1	ON	Surgery	Yes	4	4	A
Bilgin et al. [40]	2014	4.c	45	F	2	ON	Chemoradiotherapy	Yes	2	12	D
			45	F	1	ON	Observation/steroids only	No	24	24	A
			31	M	1	ON	Radiation	No	19	19	A
Caignard et al. [41]	2014	4.c	74	M	4	ON, OC	Chemoradiotherapy	Yes	12	15	D

			74	F	4	OC, OT	Chemoradiotherapy	Yes	9	11	D
Colpak et al. [42]	2014	4.d	47	M	4	OC, OT	Observation/steroids only	Yes	0.7	0.7	D
Della Puppa et al. [43]	2014	4.d	42	F	1	ON	Surgery	No	3	3	A
Kim [44]	2014	4.d	54	M	4	OC, OT	Observation/steroids only	Yes	2.3	2.3	D
Pecen et al. [45]	2014	4.d	61	F	4	ON, OC, OT	Surgery	Yes	10	10	D
Sarkar et al. [46]	2014	4.d	31	F	4	ON, OC	Chemotherapy	Yes	3	3	D
Theeler et al. [47]	2014	4.c	27	F	N/A	OC	Chemoradiotherapy	No	36	36	A
Ashur-Fabian et al. [48]	2013	4.d	64	M	4	ON, OC, OT	Radiation	Yes	27	50	D
Jiang et al. [49]	2013	4.d	67	F	4	ON, OC	Observation/steroids only	Yes	5	5	D
Liu et al. [50]	2013	4.d	70	M	3	ON, OC	Surgery	Yes	1	1	D
Manojlovic Gacic et al. [51]	2012	4.d	55	F	1	ON	Surgery	No	72	72	A
Shriver et al. [52]	2012	4.c	22	F	N/A	ON	Surgery	No	128	128	A
Matloob et al. [53]	2011	4.d	63	F	4	ON, OC	Chemotherapy	Yes	1	6	D
Simao et al. [54]	2011	4.d	62	M	2	ON, OC, OT	Chemoradiotherapy	Yes	3	12	D
Chacko et al. [55]	2010	4.d	48	M	3	ON, OT	Chemoradiotherapy	Yes	11	11	D
Pasol et al. [56]	2010	4.d	75	M	1	ON	Observation/steroids only	Yes	7	7	A
Kawasaki [57]	2009	4.d	53	F	High Grade	OC, OT	Observation/steroids only	Yes	0.7	7	D
Wu-Chen et al. [58]	2009	4.c	62	M	3	ON, OC	Chemotherapy	Yes	76	76	D
Abou-Zeid et al. [59]	2008	4.d	56	M	4	OC, ON	Radiation	Yes	3	3	D
Dinh et al. [60]	2007	4.d	48	F	4	ON	Surgery	Yes	3	13.7	D
Miyamoto et al. [61]	2006	4.d	51	M	3	ON, OC	Surgery	Yes	6	18	D
Sharif et al. [62]	2006	4.c	34	F	1	ON, OC	Chemoradiotherapy	N/A	N/A	139.2	A
			21	M	1	ON, OC	Surgery	Yes	271.2	271.2	D
			22	F	1	ON, OC	Radiation	N/A	N/A	105.6	A
Danesh-Meyer et al. [63]	2005	4.c	70	F	N/A	ON	Chemoradiotherapy	Yes	10	10	D
			60	M	3	ON	Surgery	Yes	2	20	D
			77	F	3	ON	Radiation	Yes	18	24	D
Kwon et al. [64]	2005	4.c	20	M	N/A	ON	Surgery	No	10	10	A
Albayrak et al. [65]	2004	4.d	52	F	1	OT	Surgery	No	5	5	A
Chernov et al. [66]	2004	4.d	21	F	2	OC	Surgery	No	36	36	A
Wabbels et al. [67]	2004	4.d	61	F	3	ON	Surgery	Yes	10	12	D
Murphy et al. [68]	2003	4.d	44	M	3	ON	Radiation	Yes	12	24	D
Yokoyama et al. [69]	2003	4.d	33	F	2	ON	Surgery	No	2.3	2.3	A
Synowitz et al. [70]	2002	4.d	68	M	3	ON, OC	Radiation	Yes	0.5	0.5	D
Colosimo et al. [71]	1999	4.d	20	F	1	ON, OC	Surgery	No	60	60	A
Dario et al. [72]	1999	4.d	68	F	3	OC	Surgery	Yes	6	6	D
Friedman et al. [73]	1998	4.d	78	F	High Grade	ON	Observation/steroids only	N/A	N/A	3	A
Brodovsky et al. [74]	1997	4.d	49	M	3	ON, OC, OT	Radiation	Yes	6	15	D
Liauw et al. [75]	1996	4.d	41	M	N/A	ON	Surgery	No	4	4	A
Lim et al. [76]	1996	4.d	47	F	2	ON	Surgery	No	24	24	A
Millar et al. [77]	1995	4.d	60	M	3	ON	Surgery	Yes	6	11	D
Woiciechowsky et al. [78]	1995	4.d	76	M	4	ON, OT	Surgery	Yes	1.4	1.4	D
Taphoorn et al. [79]	1989	4.d	59	F	High Grade	ON	Radiation	Yes	6	6	D
Topliss [80]	1989	4.d	19	F	N/A	ON	Surgery	No	144	144	A
Wulc et al. [81]	1989	4.c	64	F	3	ON	Surgery	Yes	48	60	A

			26	F	3	ON	Surgery	No	60	60	A
			31	F	1	ON, OC	Surgery	Yes	84	108	A
Albers et al. [82]	1988	4.c	51	M	4	ON, OC	Radiation	Yes	12	20	D
			40	F	3	OC	Radiation	No	7	7	A
Hufnagel et al. [83]	1988	4.d	41	M	3	OC	Chemotherapy	Yes	95	98	D
Ramani et al. [84]	1988	4.d	25	F	N/A	ON	Surgery	No	1	1	A
Svitra et al. [85]	1986	4.c	30	F	N/A	ON	Radiation	Yes	48	48	A
Horwich et al. [86]	1985	4.c	20	F	2	ON, OC	Surgery	No	204	204	A
			23	M	1	ON, OC	Surgery	No	120	120	A
			39	M	1	ON, OC	Surgery	No	108	108	A
			32	M	1	ON, OC	Surgery	No	120	120	A
			56	M	1	ON, OC	Surgery	No	216	216	A
Barbaro et al. [87]	1982	4.d	26	M	4	ON, OC	Surgery	Yes	8	8	D
Borit et al. [88]	1982	4.c	18	M	1	ON, OC	Radiation	N/A	N/A	150	A
			40	M	1	ON, OC	Radiation	No	120	120	A
Shapiro et al. [89]	1982	4.d	60	M	2	ON	Surgery	Yes	2.46	2.46	D
Kupersmith et al. [90]	1981	4.c	44	M	High Grade	OC	Surgery	N/A	N/A	5	A
			24	F	High Grade	OC	Surgery	N/A	N/A	24	A
			37	M	High Grade	OC, OT	Surgery	N/A	N/A	6	A
			33	M	High Grade	ON, OC	Surgery	N/A	N/A	12	A
Dosoretz et al. [91]	1980	4.c	18	F	N/A	ON, OC, OT	Surgery	N/A	N/A	72	A
			24	M	N/A	OC	Surgery	N/A	N/A	420	A
			40	M	N/A	OC	Surgery	N/A	N/A	96	A
Spoor et al. [92]	1980	4.c	69	F	3	ON, OC	Radiation	Yes	8	8	D
			64	F	3	ON, OC	Radiation	Yes	9	9	D
			59	M	3	ON, OC	Observation/steroids only	Yes	6	6	D
			64	M	3	ON	Radiation	Yes	6	8	D
			60	F	3	ON	Radiation	Yes	4	4	D
Wright et al. [93]	1980	4.c	20	F	1	ON	Surgery	No	48	48	A
Enoksson et al. [94]	1978	4.c	35	M	N/A	ON	Surgery	N/A	N/A	2.5	A
Harper et al. [95]	1978	4.c	63	F	High Grade	ON, OC, OT	Surgery	Yes	1	1	D
			75	M	4	ON, OC, OT	Observation/steroids only	Yes	1	1	D
			79	F	4	ON, OC, OT	Observation/steroids only	Yes	1	1	D
Harter et al. [96]	1978	4.c	83	M	2	ON, OC	Surgery	Yes	12	12	D
Lowes et al. [97]	1978	4.c	18	F	N/A	ON, OC	Surgery	N/A	N/A	7	A
			21	M	N/A	OC	Surgery	N/A	N/A	113	A
			24	M	1	ON, OC	Surgery	N/A	N/A	35	A
Manor et al. [98]	1976	4.d	70	M	4	ON, OC, OT	Observation/steroids only	Yes	4	4	D
Miller et al. [99]	1974	4.c	20	M	N/A	OC	Surgery	No	12	12	A
			42	M	N/A	OC	Surgery	No	72	72	A
			50	M	N/A	OC	Surgery	Yes	72	72	D
			59	M	N/A	OC	Surgery	Yes	0.1	0.1	D
			67	F	N/A	OC	Surgery	Yes	0.1	0.1	D
			19	M	N/A	ON	Surgery	No	84	84	A
			31	F	N/A	ON	Surgery	Yes	0.1	0.1	D
			47	M	N/A	ON	Surgery	No	120	120	A
			18	M	N/A	OC	Surgery	No	36	36	A
			32	F	N/A	OC	Surgery	No	36	36	A
			32	F	N/A	OC	Surgery	No	60	60	A
			40	F	N/A	OC	Surgery	No	60	60	A
Gibberd et al. [100]	1973	4.d	40	F	2	OC	Observation/steroids only	Yes	1	1	D
Hamilton et al. [101]	1973	4.d	50	M	3	ON, OT	Radiation	Yes	6	12	D
Hoyt et al. [102]	1973	4.c	46	M	3	ON	Radiation	Yes	0.53	0.53	D

			57	M	3	ON	Radiation	Yes	0.1	0.1	D
			55	M	3	ON, OC, OT	Radiation	Yes	9	9	D
			55	M	3	OC, OT	Surgery	Yes	9	9	D
			42	M	3	ON, OC	Radiation	Yes	6	6	D
Spencer [103]	1972	4.c	46	M	High Grade	ON, OC	Radiation	Yes	0.5	0.5	D
Otenasek et al. [104]	1968	4.d	51	M	N/A	ON	Surgery	No	52	52	A

Abbreviations**:** Yr= year, N/A= not available, ON= optic nerve, OC= optic chiasm, OT= optic tract, PFS= progression-free survival, Mo= months, A= alive, D= deceased. Background shading is used to group patients originating from the same study for clarity.
